# Screening of Naturally Grown European Cranberrybush (*Viburnum opulus* L.) Genotypes Based on Physico-Chemical Characteristics

**DOI:** 10.3390/foods11111614

**Published:** 2022-05-30

**Authors:** Ayşen Melda Çolak, Kerem Mertoğlu, Fatma Alan, Tuba Esatbeyoglu, İbrahim Bulduk, Erten Akbel, Ibrahim Kahramanoğlu

**Affiliations:** 1 Department of Horticulture, Faculty of Agriculture, Usak University, 64000 Usak, Turkey; aysenmelda.colak@usak.edu.tr; 2 Department of Horticulture, Faculty of Agriculture, Eskisehir Osmangazi University, 26160 Eskisehir, Turkey; 3 Department of Food Development and Food Quality, Institute of Food Science and Human Nutrition, Gottfried Wilhelm Leibniz University Hannover, Am Kleinen Felde 30, 30167 Hannover, Germany; 4 Department of Horticulture, Faculty of Agriculture, Erciyes University, 38280 Kayseri, Turkey; fatmaalan1979@gmail.com; 5 Faculty of Health Science, Uşak University, 64000 Uşak, Turkey; ibrahim.bulduk@usak.edu.tr (İ.B.); erten.akbel@usak.edu.tr (E.A.); 6 Faculty of Agricultural Sciences and Technologies, European University of Lefke, Gemikonağı, Northern Cyprus, Via Mersin 10, 99780 Karavostasi, Turkey; ibrahimcy84@yahoo.com

**Keywords:** antioxidant activity, secondary plant metabolites, organic acid, vitamin C, selection

## Abstract

It has become very important to offer species with high nutritional value as fresh or processed products for human consumption in their daily diet for balanced nutrition. In the scope of this study, 15 naturally grown European Cranberry bush (ECB) genotypes that naturally grown were characterized in terms of horticultural characteristics. Fruit length, fruit width, fruit weight, the number of fruits per each cluster and cluster weight were determined within the ranges of 8.78–10.96 mm, 7.93–10.84 mm, 0.21–0.70 g, 31–121, and 7.70–66.67 g, respectively. Ranking of the average values of examined organic acids obtained from all genotypes found as; malic acid (11,419 mg L^−1^) > citric acid (1926 mg L^−1^) > ascorbic acid (581 mg L^−1^) > oxalic acid (561 mg L^−1^). Total phenolic content (TPC) and total flavonoid content (TFC) were found at high levels in ECB with 2922–3475 mg gallic acid equivalent (GAE) L^−1^ and 1463–3163 mg quercetin equivalents (QE) L^−1^, respectively. While pomological characteristics were found to be highly positive correlated with each other, they were negatively correlated with chemical properties. Low pH was found to be an important parameter to obtain higher amounts of phytochemicals such as TPC, TFC, organic and phenolic acids correlated with strong antioxidant effects. The obtained results will be useful for both germplasm enrichment and cultivation.

## 1. Introduction

Contrary to the rapidly increasing world population, the existence of arable land is gradually decreasing [1]. This situation brings with it the necessity of expanding the cultivation of productive species with high nutritional value. For this reason, studies on the nutritional and commercial values of functional fruits especially berries such as chokeberry, bilberry, blackcurrant, blueberry, cranberry and European cranberrybush (ECB), which are widely found in nature as a wild have increased considerably recently [2,3,4,5,6].

ECB is more common in places where continental climate prevails. Plant parts of ECB such as fruits, seeds, and leaves have a high and diverse biochemical content and it is stated that it has potential in the medical field (for example, remedying of stomach or uterine bleeding, kidney problems, tuberculosis, liver diseases, diabetes, etc.) due to its high antioxidant effects [7,8]. In addition, it has been stated that it has a significant phytoremediation effect in preventing air and soil pollution, such that its cultivation should be expanded in terms of ecosystem balance in our era of intense industrial activity [9,10]. In this context, it is critical that genotypes with superior traits in today’s genetic diversity are selected and assessed according to proper consumption methodologies. In the long term, it is necessary to develop new genotypes with high commercial value by using these genotypes.

Physico-chemical properties have low heritability and high sensitivity to environmental conditions [11]. The variability of altitude causes great changes in the appearance of different climatic characteristics between regions. When the effects of other factors affecting the climate are ignored, it is observed that there are great changes in climatic characteristics depending on the change in altitude. In general, with the increase in altitude, the amount of precipitation, sun rays’ incidence angle, radiation, atmospheric layer thickness, wind activities and light intensity are increase, while the atmospheric temperature and atmospheric pressure decrease [12]. Parallel to these changes, it is determined that the physico-chemical properties of fruit species also undergo major changes [13,14]. For this reason, it is desired that the superior genotypes be developed should be obtained from a wide genetic richness so that they can perform well in combination against different ecological characteristics and stress conditions. In this context, screening and characterization of existing genetic resources has become very important.

In this study, a total of 15 ECB genotypes belonging to three different regions of Kayseri-Turkey were characterized for the first time in terms of physico-chemical properties. In addition, the question concerning how climate change due to the altitude change affects the investigated characteristics in ECB fruits was investigated. The sources of variation seen in the data were explained by factor analysis and the sensitivities of the features to these variables were explained. As a result of the correlation analyses between the features, the features which have the potential to be handled together have been determined.

## 2. Materials and Methods

### 2.1. Materials and Growing Conditions

Fruit samples from naturally grown seeds from 15 ECB genotypes were obtained from three distinct locations in Kayseri, Turkey, for this study. These materials are original and their potential has been evaluated for the first time. The altitude of study areas varied between 1266 and 1355 m. Descriptive information is presented in Table 1. Picking of fruits was carried out in October 2019 at the consumption maturity according to the sense of color and taste in all genotypes. Fruits were gathered from all sides of the plants by a single individual in order to ensure consistency in degree of maturity.

In all provinces where our study material was located, there was a harsh continental climate. Observed climatic characteristics at the selected sites during the study period are given in Table 2. Hacılar has the highest mean of year temperature. Also, the highest mean temperature values of all months were seen in Hacılar. Özvatan has the highest precipitation, while Bünyan has the lowest. But precipitation was stable in Bünyan, whereas dry periods and irregular rainfall were observed in Özvatan and Hacılar.

### 2.2. Determination of Morphological Characteristics

The collected fruits were immediately taken to the cold chain and delivered to the laboratory for analysis and measurements without wasting time. Cluster weight and fruit weight were determined using an electronic balance (Sartorius-CPA 16001S, Göttingen, Germany) with a precision of 0.001 g. A digital caliper with 0.01 mm precision was used to measure fruit width and fruit length. The quantity of fruits per cluster was calculated by counting each fruit separately in the cluster [15].

### 2.3. Preparing Samples for Analysis

The fruits left over from morphological analyzes were turned into fruit juice with an extractor. Then, juices were filtered by using coarse filter paper and stored at −20 °C until the analyzes. These juices were used for all phytochemical analyzes.

### 2.4. Determination of Soluble Solid Content (SSC) and pH

Total soluble solids (TSS) in juices were measured using a digital refractometer (Atago PR-32, Tokyo, Japan) and results are given as percentages. The pH of the juices was measured using a pH meter (Hach Co., Loveland, CO, USA) [16].

### 2.5. Spectrophotometric Assays

Total phenolic contents (TPC) of fruit juices were determined using the Folin-Ciocalteu method [17]. 200 µL of freshly crushed and filtered juice was transferred to a test tube with a volume of 10 mL. A total of 500 µL of Folin-Ciocalteu reagent was added, which had been diluted 10 times with water. The tube and its contents were left in a dark area for 5 min and then 1000 µL of sodium carbonate solution (7.5%) was added. The caps of the tubes were closed, shaken, and again kept in the dark for 1 h. The absorbance value of each sample was measured at a wavelength of 765 nm in a spectrophotometer device. The mg GAE L^−1^ value corresponding to the absorbance value was determined from the calibration graphic created using gallic acid standard solutions.

The aluminum chloride colorimetric technique was used to determine the total flavonoid content (TFC) in fruit juices [18]. 50 μL of juice, 950 μL of methanol and 6400 μL of deionized water were transferred to 10 mL tubes, then 300 μL of sodium nitrite solution (5%) was added. Then 300 μL of aluminum chloride solution (10%) was added to the mixture and it was left for 5 min, then 2000 μL sodium hydroxide solution (4%) was added to the mixture. The tube and its contents were left to stand for 15 min. The absorbance values of the samples at 510 nm were measured in a spectrophotometer. Total flavonoid contents were calculated as mg quercetin equivalent per liter from the calibration curve created with quercetin standard solutions.

Radical scavenging activity of genotypes were determined by using the DPPH (2,2-diphenyl-1-picrylhydrazyl) method with slight modifications [19]. 300 µL juice was used in 10 mL tube mixed with 5700 µL DPPH working solution with a 40 mg L^−1^ concentration. Tubes were kept in dark for 1 h to complete the reaction. Subsequently, the absorbance of this solution was measured at a wavelength of 515 nm using a spectrophotometer. The antioxidant activity value was calculated taking advantage of a decrease in the absorbance value using the following formula: Antioxidant activity (%) = (A_0_ − A_1_)/A_0_ × 100, where A_1_ is the absorbance of the mixture containing the sample and A_0_ is the absorbance value of the control solution without sample.

### 2.6. Analysis of Organic Acid Content

Samples were firstly shaken for 1 h and centrifuged at 14,000 rpm for 15 min. The supernatant was filtered using a 0.45 µm membrane filter. The filtered juice was analyzed by a HPLC device using an Agilent 1260 liquid chromatographic system (Palo Alto, CA, USA) equipped with Chemstation software, quaternary pump, autosampler, UV detector to determine the organic acids. HPLC analyses were performed using an ACE-C18 column (4 mm × 150 mm, 5 µm). The mobile phase consisted of 10 mM aqueous solution of potassium phosphate (pH 2.2 with *ortho*-phosphoric acid) with a flow rate of 1 mL min^−1^. The detector was set to 245 nm for ascorbic acid and 210 nm for all other organic acids [20].

### 2.7. Determination of Phenolic Compounds

Phenolic compounds were determined using Agilent brand 1260 model HPLC with a UV detector. An ACE-C18 (4.6 mm × 150 mm, 5 μm) column was used for chromatographic separation. The mobile phase flow rate was kept constant at 1.0 mL min^−1^. Mobile phase A was ultrapure water containing 0.1% acetic acid, while mobile phase B was acetonitrile containing 0.1% acetic acid. The gradient conditions were as follows: 0–3.25 min, 8–10% B; 3.25–8 min, 10–12% B; 8–15, 12–25% B; 15–15.8 min, 25–30% B; 15.8–25 min, 30–90% B; 25–25.4 min, 90–100% B; and 25.4–30 min, 100% B. The injection volume was 10 µL and the column temperature was kept constant at 25 °C. Detection wavelengths were chosen considering the wavelengths at which the phenolic compounds to be analyzed had maximum absorption. Syringic acid, protocatechuic acid and gallic acid were detected at 280 nm. Vanillic acid was detected at 225 nm. Coumaric acid was detected at 305 nm. Caffeic acid and chlorogenic acid and were detected at 330 nm [21].

### 2.8. Statistical Analysis

The experiment was conducted accordingly randomized plot experimental design. One-way ANOVA procedure was used in Minitab-17 for the analysis. Differences between genotypes were revealed by using Tukey multiple comparison test (*p* < 0.05). Relations among the investigated characteristics were revealed as a result of correlation analysis and expressed with Pearson correlation coefficients. Further, variation sources, all factors and characteristics relations were determined by principal component analysis (PCA) [22].

## 3. Results

General descriptive statistics of the examined properties are given in Table 3. All analyzed parameters, except for antioxidant activity, showed statistically significant differences between genotypes. Ascorbic acid (CV = 68.67%) and citric acid (CV= 64.94%) showed the highest variation among genotypes, while the lowest variation was obtained for antioxidant activity (CV = 0.60%) and TPC (CV = 5.16%). Fruit length, fruit width, fruit weight, the number of fruits per cluster and cluster weight were determined within the ranges of 8.78–10.96 mm, 7.93–10.84 mm, 0.21–0.70 g, 31–121, 7.70–66.67 g, respectively.

Malic acid was found to be the dominating organic acid in all genotypes studied, ranking of the average values of examined organic acids obtained from all genotypes found as; malic acid (11 419 mg L^−1^) > citric acid (1926 mg L^−1^) > ascorbic acid (581 mg L^−1^) > oxalic acid (561 mg L^−1^). In terms of phenolic acids, a single dominant phenolic compound did not come to the fore and genotypes showed different characteristics from each other in terms of this feature. However, in general terms, chlorogenic acid (27.13 mg 100 mL^−1^) and gallic acid (23.20 mg 100 mL^−1^) amounts are high, caffeic acid (17.76 mg 100 mL^−1^), protocatechuic acid (14.53 mg 100 mL^−1^) and vanillic acid (13.13 mg 100 mL^−1^) amounts are medium and coumaric acid (8.61 mg 100 mL^−1^), and syringic acid (8.33 mg 100 mL^−1^) amounts are low.

PCA was performed to get extended information about the distribution of genotypes by trait, correlations between morphological and biochemical characters and identification of sources of variation. This method is adapted to many small fruit species, such as strawberries [23], goji berries [13], blackcurrants [24], and even ECB [25].

According to the PCA findings, the observed variation might be explained as 72.10% by the first four components (Table 4). PC1 accounted for 35.42% of the total variance and was significantly correlated with fruit width, fruit length, fruit weight, soluble solid content, TPC, TFC, malic acid, antioxidant activity, oxalic acid, coumaric acid, antioxidant activity, chlorogenic acid and ascorbic acid. Six characters, including fruit number per cluster, cluster weight, pH, protocatechuic acid, vanillic acid, and citric acid, were placed in PC2 and accounted for 16.70% of the total variance (Table 4). It points that the determined traits in PC2 and PC1 are the most distinctive traits that separate the genotypes. PC3 and PC4 have inserted three characters, including gallic acid, syringic acid (PC3), and caffeic acid (PC4).

**Figure 1:**
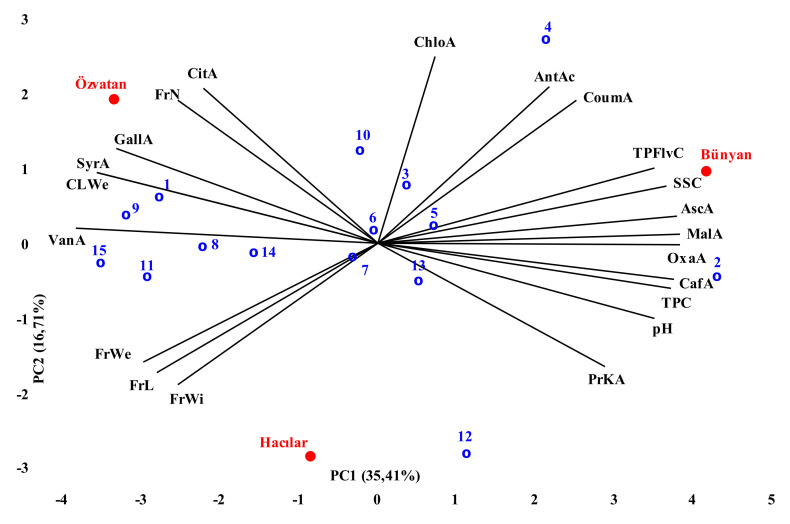
Biplot of first two PCs according to locations, genotypes and quality parameters (ChloA: Chlorogenic acid, AntAc: Antioxidant activity, CoumA: Coumaric acid, TFlvC: Total flavonoid content, SSC: Soluble solid content, AscA: Ascorbic acid, MalA: Malic acid, OxaA: Oxalic acid, CafA: Caffeic acid, TPC: Total phenolic content, PrKA: Protocatechuic acid, FrWi: Fruit width, FrL: Fruit length, FrWe: Fruit weight, VanA: Vanilic acid, CLWe: Cluster weight, SyrA: Syringic acid, GallA: Gallic acid, FrN: Fruit number, CitA: Citric acid).

PCA is a method that allows to see relations among genotypes, properties, and factors on a two-dimensional graph. Distribution of 15 genotypes according to all examined characteristics by using PCA is given in Figure 1. According to the results obtained, due to the various features that distinguish the genotypes (see Table 1) from each other, it could be concluded that they can be used for different purposes. For example, genotype 2 and 4 supplied from Bünyan are appropriate for industrial use due to their higher organic acids and SSC. Since organic acids restrict the activity of microorganisms that cause decay in crops and higher SSC increases efficiency during processing. Due to their high TFC concentration, which gives the fruit a bitter and astringent flavor, these genotypes have the potential to improve the flavor and biochemical content of fermented products such as wine and vinegar. Genotypes 7, 8, 11, and 15 came front with their morphological properties and they are more suitable for fresh consumption. Thus, it could be said that the use of these genotypes as a parental cultivar might be promising to develop new genotypes that has larger fruits with high bioactive compounds (which is the main breeding objective of ECB).

**Figure 2:**
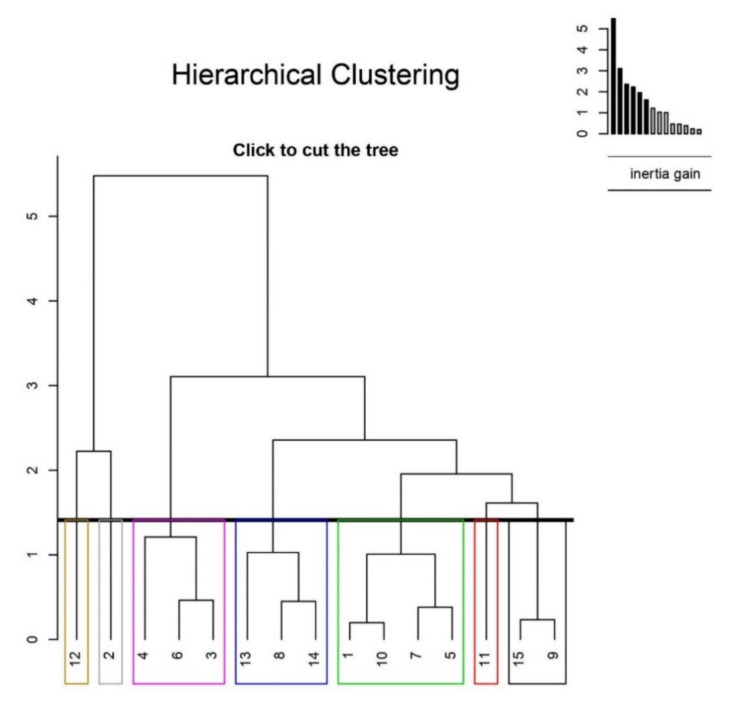
Hierarchical clustering of ECB genotypes (Numbers in different colors 1 to 15 represent the genotypes).

According to the cluster analyze shown in Figure 2, the population is grouped under two main clusters. Genotypes 2 and 12 formed cluster 1. These genotypes differ markedly from the others with their lower pH and higher TPC. Genotypes 3, 4, and 6 constitute a bar of cluster 2 and placed among these groups in terms of investigated characteristics. The members of the second branch of the second cluster stand out mainly with their pomological characteristics.

**Figure 3:**
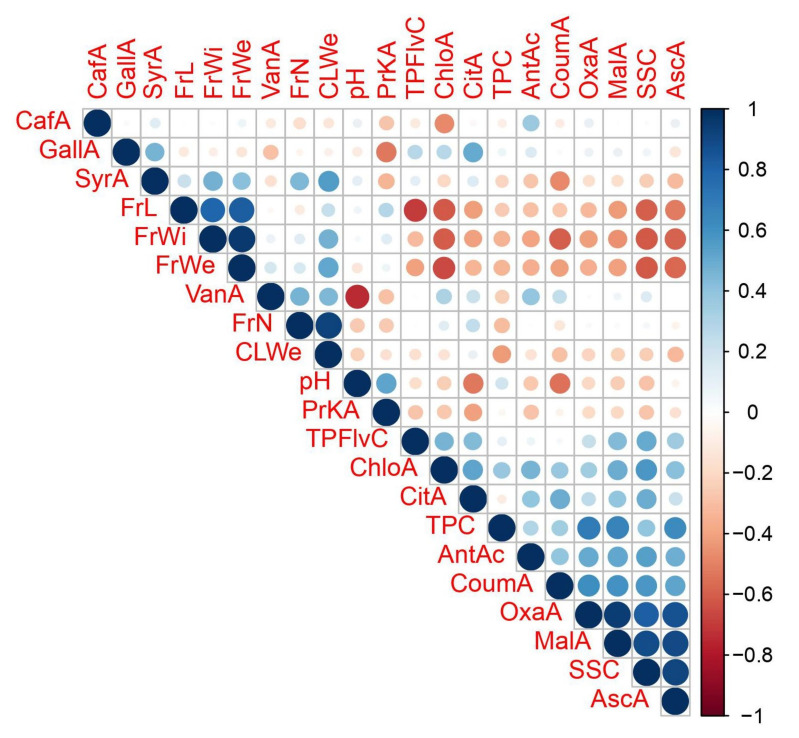
Pearson correlation coefficients among characteristics (Blue color represents a positive correlation and higher darkness and larger circles means stronger positive correlation; the same applies in red but red represents negative correlation).

Results related with correlations are given in Figure 3. The correlation coefficient among fruit length and fruit width was determined as 79 **. After fertilization, primarily cell number is increases before cell enlargement. Throughout the cell enlargement stage, the fact that longitudinal and horizontal growth takes place simultaneously explains the high correlation among these features. Since the increase in fruit size directly affects the weight, strong positive correlations were revealed among fruit weight and both fruit length and fruit width respectively at 0.83 *** and 0.95 ***. As the increase in fruit size increases the intercellular spaces that cause a decrease chemical accumulation in unit area, negative correlations were detected between all pomological features and TPC, TFC, AntAc and SSC. In addition, pomological characteristics were found in negative relations with most of individual chemical characteristics, especially those found in higher levels in fruit (Figure 3).

The degradation of organic acids carrying H^+^ ions causes an increase of the pH value. So, generally negative correlations were observed among individual phenolic and organic acids due to their acidic character with pH. It has been reported by many studies that phenolic and organic acids have antioxidant effects [26,27]. The results of the study showed parallelism with that case. Both most of individual chemicals and characteristics that express the sum of these chemicals such as TPC and TFC were seen in a positive correlation with AntAc. On the other hand, syringic and vanilic acids were found in a negative relation with TPC although they are phenolic compounds. This might be due to the fact that these chemicals turn into one another. Similar tendencies have been seen in several fruit species [28,29].

## 4. Discussion

In selection studies where genetic richness is very high, higher variations are reported for investigated characteristics in many minor fruit species such as barberry [30], rosehip [31], and ECB [32], as in our study results.

In previous researches carried out with different ECB genotypes, mean fruit length, fruit width and fruit weight properties were notified respectively as 10.18 mm, 9.71 mm, 0.65 g [33] and 11.20 mm, 15.80 mm, 0.87 g [34]. Higher ranges were reported for fruit number per cluster and cluster weight, in line with current study. Ranges of variation for these traits are notified as 29–71 and 16.7–37.6 g per cluster, respectively. [15,32,35,36,37]. Soluble solid content was found to be 11.5% on average. Similarly, it was reported as 10.4% by Sağlam [38] and 11.3% by Arslan et al. (2017) [39]. The pH of juices obtained from ECB fruits vary between 2.76 and 3.34 [36,40,41]. In our analysis, the average pH value of fruit juices was determined as 3.05 and it is within this range. Finding malic acid as a main organic acid is compatible with [42,43] but in contrast with [15,35] who reported tartaric acid as the main organic acid of ECB. Also, [37] reported that the dominant organic acid is not precise and adhere to the genotype. They defined malic acid as main in four of the ten genotypes they investigated and tartaric acid in the other six.

ECB is a species with high phenolic compounds that are important in terms of biological activity [44,45]. By [14], gallic acid, syringic acid, *p*-coumaric acid, protocatechuic acid, caffeic acid and vanillic acid amounts of ECB fruits were found 108.3 mg 100 g^−1^, 30.29 mg 100 g^−1^, 13.91 mg 100 g^−1^, 20.93 mg 100 g^−1^, respectively. Chlorogenic acid was found to be the dominant phenolic of juices belonging to five different genotypes among investigated total. Its content varied from 0.54 mg mL^−1^ to 6.9 mg mL^−1^. In the same study, TPC was also quantified and the change interval for this parameter was reported between 5.47 mg GAE g^−1^ and 10.61 mg GAE g^−1^ [46]. The current study findings were found to support these statements. TPC and TFC amounts ranged from 2922 to 3475 mg GAE L^−1^ and from 1463 to 3163 mg quercetin equivalent L^−1^, respectively. Organic and phenolic acids are defined as compounds with high antioxidant effects. ECB fruits contain these compounds at high levels. This allows the antioxidant activity to be high. As a matter of fact, the average antioxidant activity of the genotypes was found to be 84.49%. While this result was found to be compatible with the literature, the authors of [32] reported that not only the fruits of ECB but also different plant organs such as leaves, seeds etc. have a high antioxidant effect.

The obtained physicochemical results are generally consistent with earlier research [15,25,43]. Although the differences are thought to be mainly caused by the variation of the investigated genotypes, differences in climate and soil characteristics, altitude and the geographical situation of the area where the selection is made, proximity to water mass, maturity stage, harvest time and type, process, storage etc. cause serious variation on the last state of the physico-chemical properties [13,14].

## 5. Conclusions

As a result of the study, the investigated characteristics showed great variations among ECB genotypes. ECB has a potential to increase the antioxidant effect of industrial products thanks to its rich and diverse biochemical content and can prevent microorganism-induced spoilage by its low pH feature. In addition, since it is a kind of a shrub species, ECB may also be important in terms of the evaluation of female and child labor in agricultural enterprises. Outputs of current research are important with regard to increasing genetic richness. It will facilitate the selection of suitable parents in the breeding of new genotypes with superior characteristics.

## Figures and Tables

**Table 1:** Geographical description for the collection sites.

Geographical Descriptions				
Area	Latitude (N)	Longitude (E)	Altitude	Genotype Number
Özvatan	39°06′21″ N	35°41′56″ E	1266	1–5
Bünyan	38°50′44″ N	35°51′25″ E	1328	6–10
Hacılar	38°38′40″ N	35°27′03″ E	1355	11–15

**Table 2:** Climatic data of the locations in the corresponding months of the study year.

		April	May	June	July	August	September	October	Average of the Year
Temperature (°C)	Özvatan	7.2	14.8	18.8	18.8	20.1	15.7	13.6	15.5
	Bünyan	7.3	15.1	18.4	19.0	19.9	15.7	13.4	15.5
	Hacılar	8.1	16.4	20.0	20.3	21.4	17.5	15.4	17.0
Humidity (%)	Özvatan	69.7	58.3	61.1	53.9	53.1	51.8	54.5	57.4
	Bünyan	66.3	46.1	56.1	53.2	53.6	54.1	58.1	55.3
	Hacılar	71.1	51.0	60.0	53.7	53.8	50.1	55.3	56.4
Precipitation (mm.m^−^²)	Özvatan	59.2	49.8	81.2	18.4	16.8	9.3	7.7	34.6
	Bünyan	37.1	10.7	67.9	14.8	25.9	18.8	13.4	26.9
	Hacılar	35.7	20.8	78.9	36.5	4.7	9.2	19.0	29.2

**Table 3:** Descriptive statistics for physico-chemical characteristics.

Abbreviation		Unit	Minimum	Maximum	Mean ± StDev	CV (%)
General physico-chemical characteristics						
Width of fruit	FrWi	Mm	7.93	10.84	9.78 ± 0.84	8.59
Length of fruit	FrL	Mm	8.78	10.96	9.92 ± 0.70	7.08
Weight of fruit	FrWe	G	0.21	0.70	0.56 ± 0.70	24.46
Number of fruits in the cluster	FrN	number	31	121	55.57 ± 24.98	44.95
Weight of cluster	CLWe	G	7.70	66.67	31.41 ± 15.94	50.75
Soluble solid content of juice	SSC	%	7.10	24.10	11.50 ± 4.16	36.20
pH	pH	-	2.76	3.34	3.05 ± 0.17	5.65
Organic acids						
Malic acid	MalA	mg L^−1^	5215	22,026	11,420 ± 4443.28	38.91
Citric acid	CitA	mg L^−1^	512	4845	1926 ± 1251.26	64.94
Oxalic acid	OxaA	mg L^−1^	255	1064	562 ± 219.64	39.04
Ascorbic acid	AscA	mg L^−1^	202	1814	581 ± 222.33 *	68.67
Phenolic acids						
Chlorogenic acid	ChloA	mg 100 mL^−1^	23.64	30.33	27.13 ± 1.89	6.95
Gallic acid	GallA	mg 100 mL^−1^	20.21	26.59	23.20 ± 1.74	7.50
Caffeic acid	CafA	mg 100 mL^−1^	14.82	19.92	17.76 ± 1.61	9.43
Protocatechuic acid	PrKA	mg 100 mL^−1^	12.91	17.76	14.53 ± 1.46	10.03
Vanillic acid	VanA	mg 100 mL^−1^	11.57	15.05	13.13 ± 1.12	9.04
Syringic acid	SyrA	mg 100 mL^−1^	6.26	11.97	8.33 ± 1.57	16.77
Coumaric acid	CoumA	mg 100 mL^−1^	6.38	11.18	8.61 ± 1.29	16.33
Phenolic content (Total)	TPC	mg GAE L^−1^	2922	3475	3121 ± 161.08	5.16
Flavonoid content (Total)	TFC	mg QUE L^−1^	1463	3163	2332 ± 510.15	21.90
Antioxidant activity						
Antioxidant activity (DPPH)	AntAc	%	83.49	85.36	84.49 ± 0.51	0.60

StDev, standard deviation; *: the mean of the statistical difference between genotypes; CV, coefficient of variation.

**Table 4:** The eigenvalues of the principal component axes from PCA.

	PC1	PC2	PC3	PC4
Width of fruit	0.81 *	0.20	−0.14	0.34
Length of fruit	0.73	−0.05	−0.35	0.35
Weight of fruit	0.77	0.27	−0.28	0.38
Fruit Number per cluster	0.09	0.77	−0.07	−0.10
Cluster weight	0.41	0.76	−0.13	0.02
Soluble solid content	−0.91	0.10	−0.07	0.13
pH	0.28	−0.69	0.37	0.06
Malic acid	−0.86	0.02	−0.14	0.36
Citric acid	−0.54	0.56	0.24	−0.09
Oxalic acid	−0.78	−0.03	−0.22	0.48
Ascorbic acid	−0.85	−0.16	−0.18	0.26
Chlorogenic acid	−0.72	0.23	0.14	−0.40
Gallic acid	−0.17	0.28	0.71	0.24
Caffeic acid	0.02	−0.08	0.06	0.60
Protocatechuic acid	0.30	−0.597	−0.29	−0.30
Vanillic acid	−0.11	0.67	−0.58	−0.23
Syringic acid	0.37	0.48	0.54	0.42
Coumaric acid	−0.69	0.03	−0.43	−0.05
Phenolic content (Total)	−0.57	−0.40	−0.04	0.34
Flavonoid content (Total)	−0.51	0.19	0.48	−0.15
Antioxidant activity	−0.62	0.17	−0.21	0.23
Cumulative variance (%)	35.42	52.12	62.88	72.10

*: Values in red indicate that the feature mostly interacts under the respective PC.

## Data Availability

Not applicable.
